# Surgical repair of an aortico-left ventricular tunnel with Takayasu's arteritis

**DOI:** 10.1186/s13019-021-01746-x

**Published:** 2022-01-28

**Authors:** Runqian Sui, Jingtao Zhong, Jie Zi, Anbiao Wang

**Affiliations:** Department of Cardiovascular Surgery, Shandong Provincial Hospital Affiliated to Shandong First Medical University, No. 324 Jingwu Road, Jinan, 250021 China

**Keywords:** Aortic disease, Aortic-left ventricular tunnel, Takayasu’s arteritis

## Abstract

Aortico-left ventricular tunnel is a very rare congenital cardiac anomaly, always arises from the right coronary sinus and enters the left ventricle. However, aortico-left ventricular tunnel associated with Takayasu's arteritis has not been described so far in the literature. Here, we present an unusual case of aortico-left ventricular tunnel associated with Takayasu's arteritis in a 44-year-old man.

## Introduction

Aortico-left ventricular tunnel (ALVT) is a rare congenital heart disease, estimated for up to 0.05% of congenital heart malformations [1, 2], and approximately 130 cases have been reported so far. This malformation was first described by Levy et al. [3]. The anomaly is characterized by an abnormal paravalvar communication between the ascending aorta and the left ventricle. In most reported cases, the communication originates from the aorta above the right coronary sinus and terminates in the left ventricle below the right aortic cusp [4]. However, ALVT associated with Takayasu's arteritis has not been described so far in the literature. Here, we present an unusual case of aortico-left ventricular tunnel associated with Takayasu's arteritis (TAK) in a 44-year-old man.

**Fig. 1:**
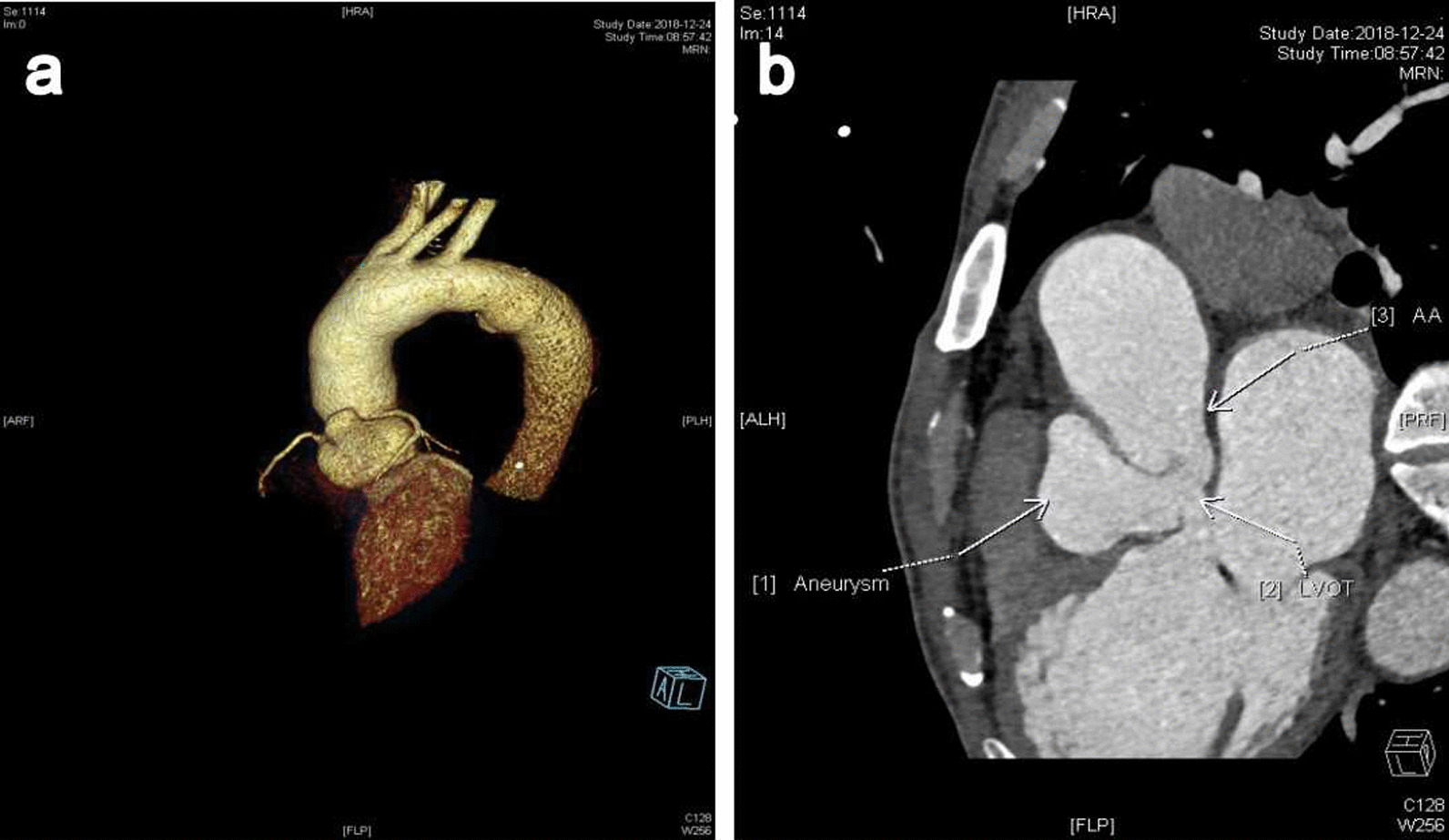
Preoperative examination. **a** Three-dimensional construction of cardiac computed tomography angiogram. **b** An aneurysmal ALVT was demonstrated with an oval entrance in the tubular aorta above the the right-coronary sinutubular ridge, draining into the LVOT. *ALVT* aorto-left ventricular tunnel, *LVOT* left ventricular outflow tract

A 44-year-old man, with progressive chest tightness, and asthma, lasting 1 week, came to the hospital. He was diagnosed with Takayasu's arteritis at local hospital two years ago. Physical examination revealed a grade 3/6 systolic and diastolic murmur maximal at the aortic valve auscultation location area along left sternal edge of the 3nd intercostal space. Cockade sign was positive according to classification criteria for TAK established by the American College of Rheumatology in 1990 [5]: claudication of extremities, decrease of brachial artery pulse, blood pressure difference > 10 mmHg. Laboratory tests showed N-terminal prohormone of brain natriuretic peptide (NT-pro BNP) of 5199 pg/mL, and C-reactive protein of 15.4 mg/L. Chest X-ray showed cardiomegaly (cardiothoracic ratio, 55%) and aortic sclerosis. ECG displayed first degree atrioventricular block with left ventricular high voltage. Transthoracic echocardiography revealed a Type IV ALVT with mild aortic regurgitation, 58% ejection fraction of the left ventricle. A capsular-like bulge was noted at the aortic root, extending from the tubular aorta above the right aortic cusp sinutubular junction, tunneling through the interventricular septum and draining into the left ventricular outflow tract (LVOT). Cardiac CTA also confirmed the findings and the diagnosis (Fig. 1a, b).

**Fig. 2:**
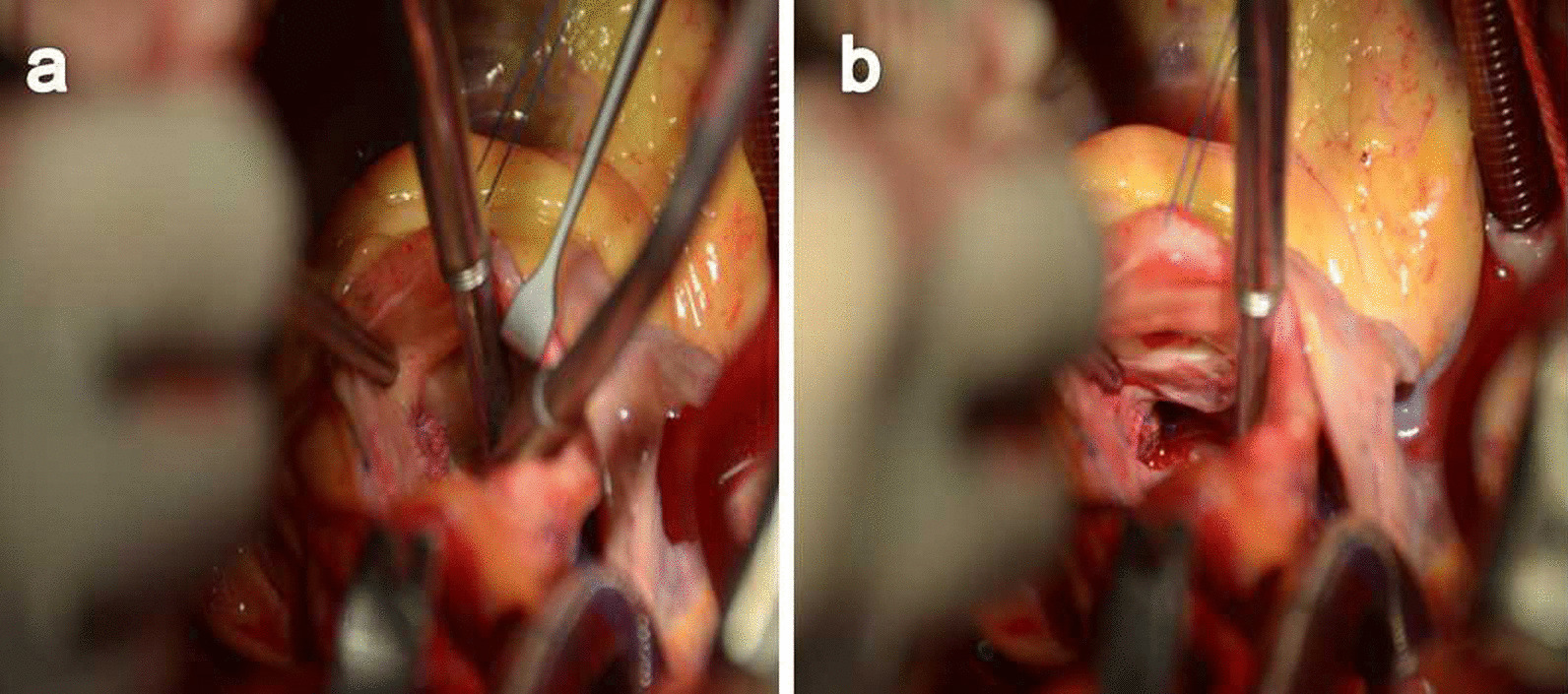
Intraoperative images. Two pieces of bovine pericardium were used to repair the aortic entrance (**a**) and left ventricular outlet (**b**) under extracorporeal circulation

**Fig. 3:**
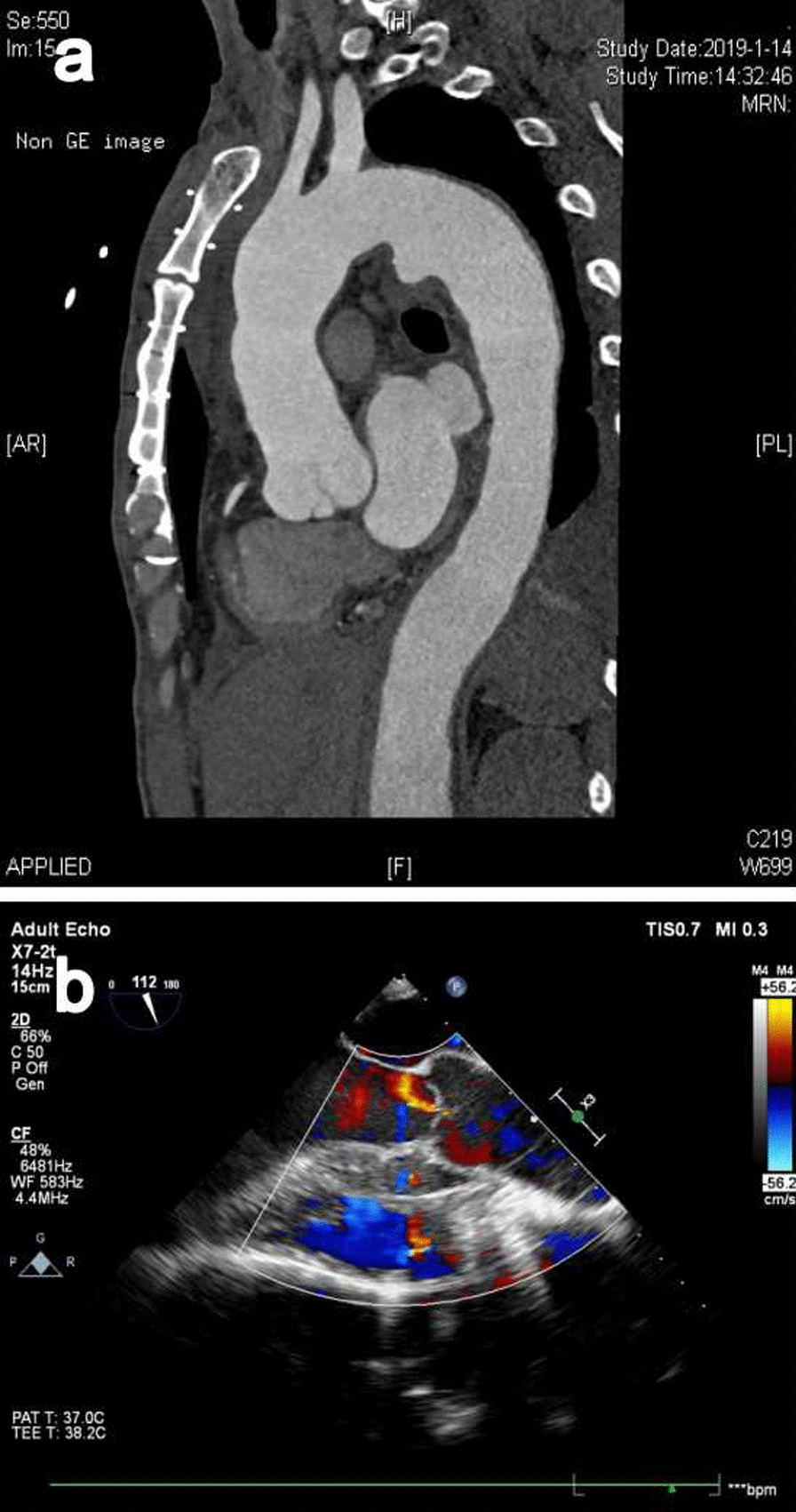
Postoperative examination. Aortic CT-scan (**a**) and Transthoracic echocardiography (**b**) image were performed and demonstrated no residual ALVT, and no aortic regurgitation

The patient was scheduled for cardiac surgery for repairing the malformation. Under general anesthesia, median sternotomy was performed. Cardiopulmonary bypass with mild hypothermia was established after heparin administration (3 mg/kg), with standard ascending aorta and right atrium cannulation. A vent was inserted into the left ventricle through the right superior pulmonary vein. The ascending aorta was cross-clamped, and myocardial protection was achieved with antegrade injection of HTK cardioplegic solution into the aortic root. The ascending aorta was incised transversely, the aortic valve was evaluated and the aortic site of the tunnel was identified. The 1.5-cm oval entrance of the tunnel was found in the tubular aorta above the right-coronary sinutubular ridge, and the margin of the tunnel was sharp with little inflammatory exudation. A probe revealed that the left ventricular end of the tunnel vented beneath the aortic valve in the interventricular septum. The morphology suggested Type IV ALVT. The tunnel defect was successfully repaired with two bovine pericardium patches to close both the aortic and ventricular orifices using a double-ended 6-0 prolene suture. We addressed the aortic valve insufficiency by correcting the height of the commissure between the left and the right coronary cusps (Fig. 2a, b). The patient was slowly weaned from cardiopulmonary bypass with dopamine for inotropic support. Heparin was reversed with protamine and routine chest closure was performed. The aortic cross-clamp time was 75 min. Cardiopulmonary bypass time was 95 min. The patient made an uneventful recovery and was discharged 8 days after operation. Aortic CT-scan and transthoracic echocardiography were performed and demonstrated no residual ALVT, and no aortic regurgitation at follow-up (Fig. 3a, b). The heart murmur and signs of aortic regurgitation disappeared. The patient is undergoing regular follow up and remains asymptomatic.

## Discussion

We present an unusual case of aortico-left ventricular tunnel associated with Takayasu's arteritis. The etiology of the ALVT malformation remains uncertain; however, there was a great deal of speculation about the etiology of the tunnel, ranging from congenital to acquired nature of the defect. Hovaguimian et al. proposed a new anatomic classification, they defined four anatomic types of ALVT, namely those with a slit-like aortic orifice with no distortion of the valve, those with a larger oval-shaped aortic orifice with an aneurysmal extracardiac component, those with an oval aortic orifice and an aneurysmal intracardiac component, and those with a combination of intracardiac and extracardiac aneurysmal formation [6, 7]. The clinical presentation is dependent on the size of the tunnel and the degree of aortic regurgitation. Chest pain, chest tightness and cardiac murmur were the major clinical symptoms. ALVT must be distinguished from other lesions, including sinus of Valsalva fistula, common arterial trunk with valvar regurgitation, aorto-pulmonary window, and ventricular septal defect with aortic regurgitation [8]. Diagnosis can usually be made by echocardiography and computed tomography. Left ventricular enlargement and hypertrophy was noted in almost all patients. The association of normal ventricular architecture, a dilated aortic root, flow through the tunnel, and identification of aortic regurgitation should suggest the diagnosis [9]. The results of transthoracic echocardiography and cardiac catheterization are here interpreted as showing a significant bidirectional turbulent flow via a tubular communication between the aortic root just above the level of the aortic sinus and the left ventricle. Cardiac computed tomography angiography can confirm the diagnosis of an ALVT by depicting the entry of the contrast material from an opening above the aortic sinus of Valsalva into the LV, and may be useful in differentiating these anomalies with greater anatomical details.

The presence of the tunnel is due to the failure of attachment of the aortic leaflet along its semilunar hinge within the aortic root [10]. The optimal management of patients with an ALVT is a prompt surgical closure of the defect after establishing the diagnosis. Although there are case reports of percutaneous device closure of the defect, surgery is the ideal and standard treatment strategy for preventing further myocardial damage and LV dilation [11]. The preferred method of surgical repair is double patch closure of both the aortic and the ventricular orifices because this provides additional protection from late valvular regurgitation and recurrence of the fistulous tract. There has been concern about possible aortic valve leaflet distortion resulting in valvular insufficiency after direct suturing at the base of the right coronary cusp to the aortic wall compared with patch closure [12]. If the aortic orifice of the ALVT is not closed, residual high pressure in the blind-ending pouch may compress the right ventricular outflow. With patch closure, utmost precautions must be taken to avoid collision or traction of the commissure between the coronary aortic valvar leaflets, since the aortic orifice is usually located just above the right sinutubular junction [9, 13].

Takayasu's arteritis is an unusual multisystem inflammatory disorder of unknown cause and is characterized by marked thickening of the aortic wall, with fibrosis of the intima and adventitia. In addition, the pathologic findings of Takayasu’s arteritis demonstrate disruption of medial elastic fibers [14]. In our patient, little inflammatory exudation was observed at the time of surgery, and the lesion was already degraded. The ALVT associated with Takayasu’s arteritis case reported here is unusual and the final diagnosis of Type IV ALVT. The tunnel was characterized as an oval entrance in the tubular aorta above the right-coronary sinutubular ridge, draining through the interventricular septum into the LVOT, which was confirmed by surgical inspection. The goal of initial repair is to prevent flow of blood through the tunnel while maintaining aortic valve function. The repair should be individualized according to the anatomy of the tunnel. When the tunnel originated in the right coronary aortic sinus, support for the leaflet is essential for retaining the aortic sinus and avoiding subpulmonary obstruction [15]. The patient performed well after surgical closure of the tunnel. Regular follow-up even after correction of the tunnel is necessary to detect any recurrence of the tunnel, aortic valve insufficiency, or aneurysmal enlargement of the ascending aorta.
